# A Multi-Classification Model for Predicting the Invasiveness of Lung Adenocarcinoma Presenting as Pure Ground-Glass Nodules

**DOI:** 10.3389/fonc.2022.800811

**Published:** 2022-04-28

**Authors:** Fan Song, Lan Song, Tongtong Xing, Youdan Feng, Xiao Song, Peng Zhang, Tianyi Zhang, Zhenchen Zhu, Wei Song, Guanglei Zhang

**Affiliations:** ^1^ Beijing Advanced Innovation Center for Biomedical Engineering, School of Biological Science and Medical Engineering, Beihang University, Beijing, China; ^2^ Department of Radiology, Peking Union Medical College Hospital, Chinese Academy of Medical Sciences and Peking Union Medical College, Beijing, China; ^3^ School of Medical Imaging, Shanxi Medical University, Taiyuan, China; ^4^ 4 + 4 MD Program, Chinese Academy of Medical Sciences and Peking Union Medical College, Beijing, China

**Keywords:** adenocarcinoma of lung, pure ground-glass nodule, computer-assisted diagnosis, neoplasm invasiveness, early diagnosis, prognosis

## Abstract

**Objectives:**

To establish a multi-classification model for precisely predicting the invasiveness (pre-invasive adenocarcinoma, PIA; minimally invasive adenocarcinoma, MIA; invasive adenocarcinoma, IAC) of lung adenocarcinoma manifesting as pure ground-glass nodules (pGGNs).

**Methods:**

By the inclusion and exclusion criteria, this retrospective study enrolled 346 patients (female, 297, and male, 49; age, 55.79 ± 10.53 (24-83)) presenting as pGGNs from 1292 consecutive patients with pathologically confirmed lung adenocarcinoma. A total of 27 clinical were collected and 1409 radiomics features were extracted by *PyRadiomics* package on *python*. After feature selection with L2,1-norm minimization, logistic regression (LR), extra w(ET) and gradient boosting decision tree (GBDT) were used to construct the three-classification model. Then, an ensemble model of the three algorithms based on model ensemble strategy was established to further improve the classification performance.

**Results:**

After feature selection, a hybrid of 166 features consisting of 1 clinical (short-axis diameter, ranked 27th) and 165 radiomics (4 shape, 71 intensity and 90 texture) features were selected. The three most important features are wavelet-HLL_firstorder_Minimum, wavelet-HLL_ngtdm_Busyness and square_firstorder_Kurtosis. The hybrid-ensemble model based on hybrid clinical-radiomics features and the ensemble strategy showed more accurate predictive performance than other models (hybrid-LR, hybrid-ET, hybrid-GBDT, clinical-ensemble and radiomics-ensemble). On the training set and test set, the model can obtain the accuracy values of 0.918 ± 0.022 and 0.841, and its F1-scores respectively were 0.917 ± 0.024 and 0.824.

**Conclusion:**

The multi-classification of invasive pGGNs can be precisely predicted by our proposed hybrid-ensemble model to assist patients in the early diagnosis of lung adenocarcinoma and prognosis.

## Introduction

At present, with the widespread clinical application of computed tomography (CT) and the popularity of early lung cancer screening, more and more ground-glass nodules (GGNs) are detected. GGN is a nodule showing hazy increased density on thin-slice CT, with preservation of bronchial and vascular margins (1, 2). According to whether there are solid components in the lesion, GGN can be further divided into pure GGN (pGGN) and part-solid GGN. The appearance of a persistent invasive pGGN may suggest a high risk of early malignant tumor, so distinguishing the invasiveness of pGGNs is critical. A pathological classification was established in 2011 with respect to the degree of invasion: atypical adenomatous hyperplasia (AAH), adenocarcinoma *in situ* (AIS), minimally invasive adenocarcinoma (MIA) and invasive adenocarcinoma (IAC) (3).

In general, the tumor doubling time of pre-invasive adenocarcinoma (PIA, namely AAH/AIS) can reach more than two years, and through partial resection, the 5-year survival rate of patients can reach 100% (4–7). For MIA, sublobectomy or lobectomy is commonly used, and the 5-year survival rate is close to 100%. For IAC, unless the lesion diameter is less than 2 cm or the ground-glass component is greater than 75%, the 5-year survival rate is only 60%-80% even if lobectomy and lymph node dissection are performed. Therefore, the preoperative differentiation of PIA, MIA and IAC appearing as pGGNs is very important for clinical decision making.

At present, the invasiveness of pGGNs is usually diagnosed clinically based on conventional qualitative and quantitative CT parameters that can be recognized by radiologists with naked eyes, such as the average CT value, lesion size, lobulation and spiculation et al. (8–11). However, the recognition of these features largely depends on the experience of radiologists, which is subjective and time-consuming. Radiomics, as an emerging technology, transforms medical images into quantitative data and then extracts many quantitative features that can be used to accurately and quickly evaluate tumor characteristics (12). It has the advantages of strong explanation and more stable performance on a large number of small-scale medical data sets. At present, it is still widely studied in the field of clinical computer-aided detection (CAD). The domain of investigation in radiomics consists of large-scale radiological image analysis and association with biological or clinical endpoints such as differential diagnosis, survival time prediction, disease metastasis prediction and so on (13–15). Many studies have confirmed that radiomics had high clinical application value in the invasiveness classification of lung adenocarcinoma manifesting as GGNs (2, 16–19). Our previous research also established an efficient clinical-radiomics model to classify the invasiveness of pGGNs (20). However, current studies mainly predicted the invasiveness of lung adenocarcinoma as invasive or non-invasive, and multi-classification studies with more clinical application value were rarely conducted to distinguish the degree of invasion in more detail.

**Figure 1:**
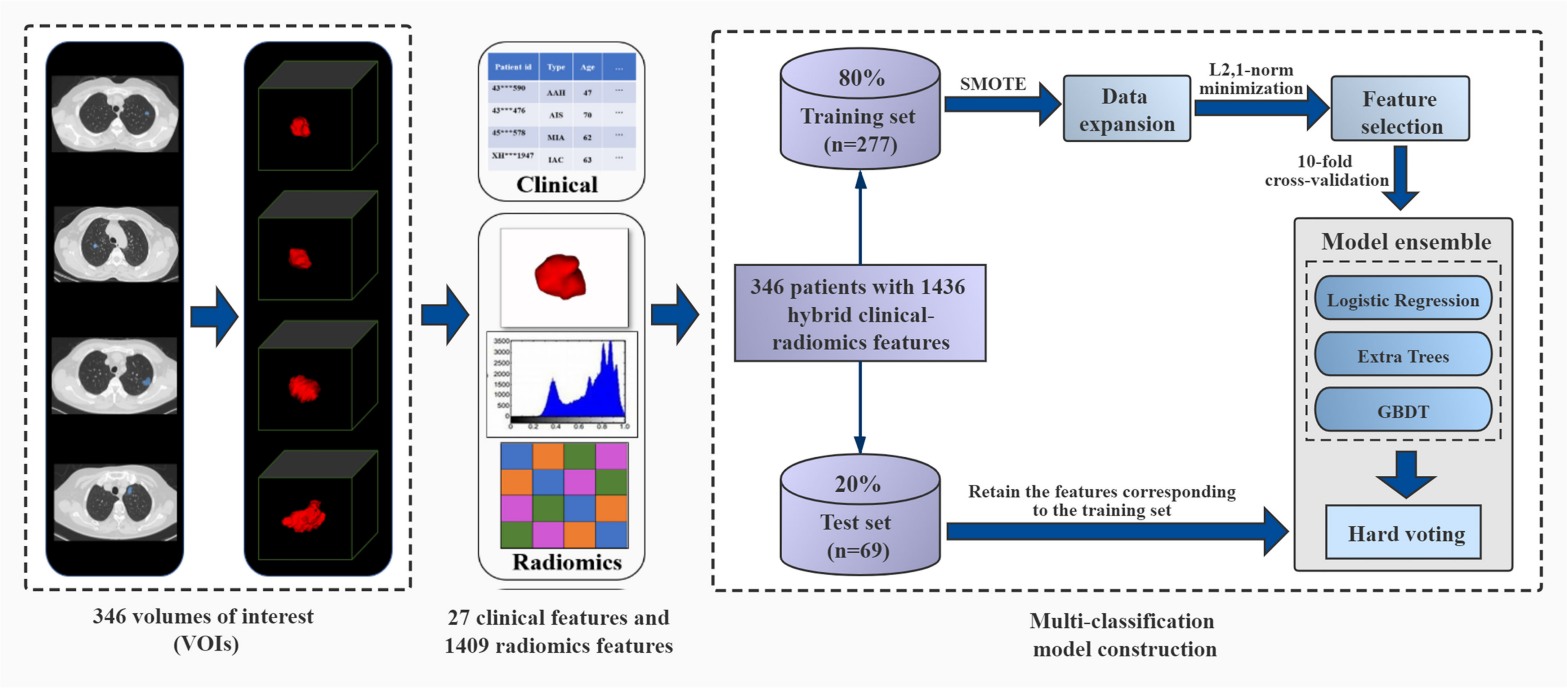
The framework of ensemble multi-classification model based on hard voting. It includes volumes of interest (VOIs) segmentation, clinical feature collection and radiomics feature extraction, division of training set and test set, data expansion on the training set, feature selection, parameter training of three models, model ensemble with the hard voting and model performance testing.

Therefore, this study aims to use quantitative imaging and clinical semantic features to establish a multi-classification radiomics model that can accurately predict different invasion grades (PIA, MIA, IAC) of pGGNs, and assist patients in the early diagnosis of lung cancer and prognosis. We used a large number of clinical features provided by radiologists and radiomics features extracted from CT images. The model ensemble strategy can integrate results obtained from multiple classifiers, and has been proven to obviously improve classification and generalization performance in various research fields (21, 22). So in this work, we introduced this strategy to integrate the classification results of three algorithms, and finally constructed a multi-classification model to effectively distinguish the degree of invasion for pGGNs. The framework of our proposed model is shown in **Figure 1**.

## Methods and Materials

### Patients

Our study was approved by the institutional review board (No. S-K1061), and informed consent was waived. This retrospective study reviewed the CT images of lung adenocarcinoma patients confirmed by the surgical pathology of Peking Union Medical College Hospital from November 2016 to August 2020. The inclusion criteria were as follows: (1) CT examination within one month before surgery; (2) isolated nodules with pure GGN Section (maximum long-axis diameter < 3 cm); (3) Tumor lesions in the clinical stage of T1N0M0. The exclusion criteria were as follows: (1) Radiotherapy or chemotherapy before CT examination; (2) pGGNs with very small size (maximum long-axis diameter < 3 mm). The demographic and clinical data (such as gender, age, smoking history, etc.) of patients were also recorded.

### Image Acquisition

Non-contrast enhanced chest CT scans were carried out using multidetector CT scanners from Siemens (Somatom Definition Flash or Somatom Force), General Electric (Discovery CT750 HD), Philips (IQon CT) or Toshiba (Aquilion 64). Breath-hold training was carried out before each examination. The following scanning parameters were used: slice thickness/slice increment 1 mm, 0.625 mm or 0.5 mm; rotation time 0.5 or 0.6 second; pitch 0.984 or 1.2; matrix 512*512; field of view (FOV): 350 mm; standard algorithm reconstruction; tube voltage 120 kVp, tube current adjusted automatically.

### Volumes of Interest (VOIs) Segmentation

The anonymized thin-slice CT images (≤1 mm, DICOM format) was delineated and segmented on lung window (window width, 1200 HU; window level, -500 HU) using ITK-SNAP (www.itk-snap.org). Two radiologists (with 15 and 4 years of experience in chest CT image interpretation) manually segmented the nodules slice by slice, both of them were blinded to the clinical data of each subject. Finally, segmentation results were output as three-dimensional VOI files (NRRD format) for subsequent feature extraction.

### Radiomics Feature Extraction

**Figure 2:**
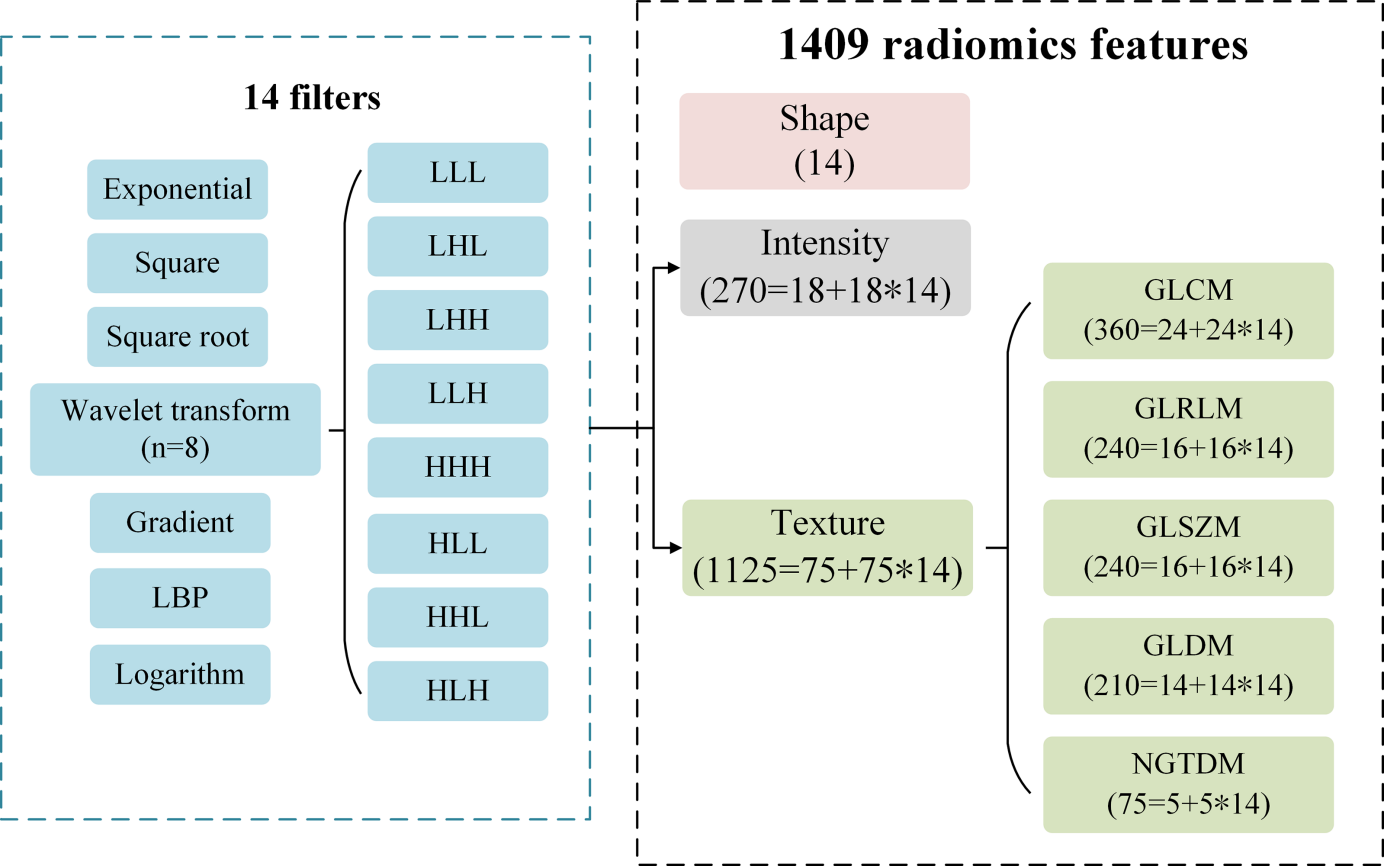
The type description of 1409 radiomics features. A total of 1409 features consisting of intensity, shape and texture features are extracted from the original images and filtered images. A total of 14 filters are used to calculate the original intensity and texture features, respectively.

A total of 1409 radiomics features were extracted from the three-dimensional VOI of each tumor by *PyRadiomics* package (version 2.1.2) (23) on *python* (version 3.7.1). We extracted three categories consisting of 1409 radiomics features (**Figure 2**): (I) Tumor shape features (n = 14). They were used to quantify the degree of regularity of tumor volume shape, and all 14 features were only from the original image. (II) Tumor intensity features (n = 270). They included 18 original image features and 252 filtered image features to describe the overall density information of each tumor volume. Each original image feature was recalculated through 14 filters, so 252 filtered image features were obtained (18 * 14 = 252). (III) Tumor texture features (n = 1125). They were used to describe the heterogeneity within the tumor volume by gray level co-occurrence matrix (GLCM, n = 336), gray level run length matrix (GLRLM, n = 224), gray level size zone matrix (GLSZM, n = 224), gray level dependence matrix (GLDM, n = 196) and neighbourhood gray-tone difference matrix (NGTDM, n = 70). Among them, there were 75 features from the original image (GLCM = 24, GLRLM = 16, GLSZM = 16, GLDM = 14, NGTDM = 5). Similar to the intensity features, original texture features were also calculated through 14 filters, and a total of 1050 filtered features were obtained (75 * 14 = 1050).

### Data Division and Expansion

In this work, a total of 346 pGGNs were randomly assigned to the training set (n = 277) and test set (n = 69) at a ratio of 8:2. Due to the existing problem of data imbalance (PIAs: MIAs: IACs = 88: 71: 118) on the training set, the synthetic minority oversampling technique (SMOTE) was used to expand and balance the number of samples (24). It is a commonly used data augmentation technology to deal with unbalanced data, by calculating the Euclidean distance between samples and then inserting new samples to original dataset automatically. On the training set, the 277 cases of three categories was expanded to 606 cases (PIAs: MIAs: IACs = 202: 202: 202), in which the ratio of the three categories was 1:1:1. The cases on the test set (PIAs: MIAs: IACs = 21: 18: 30) must maintain independence and no data expansion.

### Feature Selection

After collecting 27 clinical features and extracting 1409 radiomics features, a total of 1436 hybrid clinical-radiomics features were obtained. Since a large number of redundant features could reduce the classification effect and cause the model to be highly complex, this study used the L2,1-norm minimization (25) for feature selection. The total 1436 features were first sorted from high to low according to their importance (weight coefficients) to the classification label (26), and then the top features were selected to participate in the classification. The number of selected features was determined according to the classification results of 10-fold cross-validation (27) on the training set.

### Construction of Multi-Classification Models

In this study, we first respectively used logistic regression (LR), extra trees (ET) and gradient boosting decision tree (GBDT) algorithms to construct the three-classification model for predicting the invasiveness of pGGNs based on the selected hybrid clinical-radiomics features. Furthermore, in order to improve the classification performance, we adopted the model ensemble strategy of hard voting (22) to integrate the prediction results of the three algorithms. In addition, we also used independent clinical features and independent radiomics features to respectively construct ensemble models of the three algorithms as the comparisons. These algorithms were implemented by the scikit-learn package (version 0.23.2), and all model training process was completed on python 3.7.1. The 10-fold cross-validation and grid search were used to find optimal hyperparameters on the training set, and then the manual fine-tuning process was executed.

### Statistical Methods

The performances of all multi-classification models were quantitatively evaluated by the precision, recall, F1-score, accuracy on the training set and the independent test set:


$$Precision=\frac{TP}{TP+FN}\;(1)$$

$$Recall=\frac{TP}{TP+FN}\;(2)$$

$$F_{1}-score=\frac{2\cdot recall*precision}{\left(recall+precision\right)}=\frac{2\cdot TP}{2\cdot TP+FN+FP}\;(3)$$

$$Accuracy=\frac{TP+TN}{TP+TN+FP+FN}\;(4)$$

where *TP*, *TN*, *FP* and *FN* stand for true positive, true negative, false positive and false negative, respectively. And all evaluation metrics were performed in the *scikit-learn* package. The above evaluation indicators of multi-classification can be directly calculated through *python* (version 3.7.1). Other simple data recording and calculation were done using Excel 2016 (Microsoft Corp., Seattle, WA, USA). And the statistical significance of t-test was set at *p* < 0.05.

## Results

### The Result of Patient Screening

**Figure 3:**
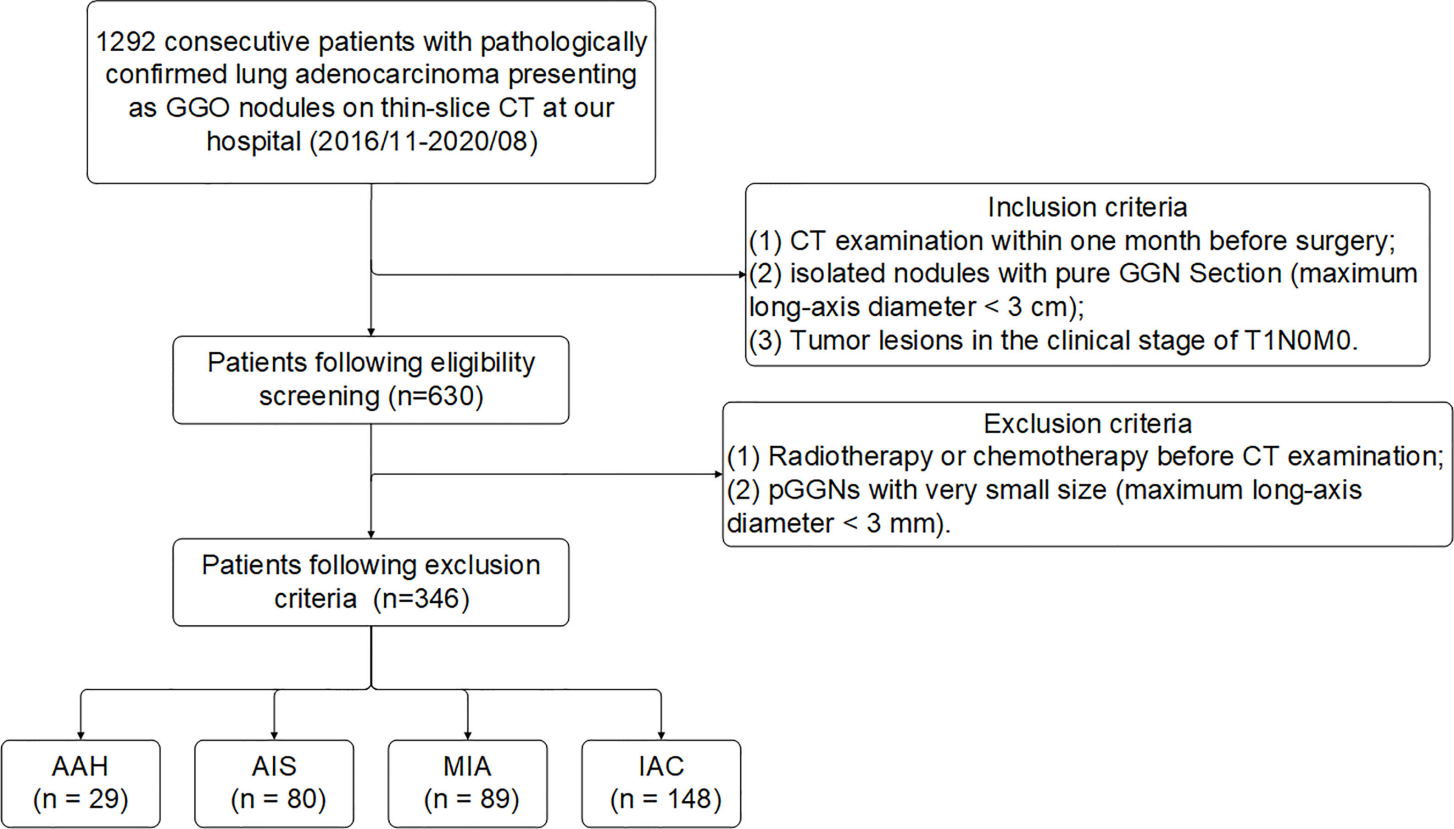
Flowchart of patient enrollment and exclusion criteria of data set. Numbers in parentheses are the numbers of pGGNs. GGO, ground glass opacity nodule.

In this study, a total of 1292 consecutive patients with pathologically confirmed lung adenocarcinoma presenting as ground glass opacity (GGO) nodules on thin-slice CT at our hospital (2016/11-2020/08) were initially collected. By inclusion criteria, 630 patients were obtained and then further screened by exclusion criteria (**Figure 3**). Finally, 346 pGGNs met the standard. All pGGNs were confirmed by experienced radiologists as AAH (n = 29), AIS (n = 80), MIA (n = 89), or IAC (n = 148).

### Patients and Clinical Features Collection

The clinical features collected by the research include 4 basic clinical features from medical records and 15 conventional CT features, as shown in **Table 1**. This study used one-hot encoding to quantitatively process clinical features. One-hot encoding is a data processing method that converts qualitative disordered data into quantitative ordered data (28). The main idea is to use multiple state registers to encode multiple states, so that each state has an independent register, and only one digit is valid at any time (29). After one-hot encoding, 19 original clinical features were converted into 27 usable features. The cases in the training set and the test set do not show significant differences in all clinical features.

**Table 1:** Clinical features of 346 patients on the training set and test set.

Clinical features	Total (n=346)	Training set (n=277)	Test set (n=69)	*p* value
**Age (years)**	55.79 ± 10.53 (24-83)	55.72 ± 10.83 (27-83)	56.06 ± 10.70 (24-76)	0.811
**Gender**				0.391
Female	297 (85.8)	240 (86.6)	57 (82.6)	
Male	49 (14.2)	37 (13.4)	12 (17.4)	
**Smoking history**				0.419
Never smoker	319 (92.2)	257 (92.8)	62 (89.9)	
Former/current smoker	27 (7.8)	20 (7.2)	7 (10.1)	
**Smoking index (pack-years)**	1.34 ± 6.18 (0-75)	1.27 ± 6.34 (0-75)	1.59 ± 5.50 (0-30)	0.698
**Lesion involved lobe**				0.764
Right upper lobe	126 (36.4)	101 (36.5)	25 (36.2)	
Right middle lobe	17 (4.9)	12 (4.3)	5 (7.2)	
Right lower lobe	62 (17.9)	51 (18.4)	11 (15.9)	
Left upper lobe	99 (28.6)	80 (28.9)	19 (27.5)	
Left lower lobe	42 (12.1)	33 (11.9)	9 (13.0)	
**Maximum long-axis diameter (mm)**	12.39 ± 5.60 (3.5-30)	12.37 ± 5.63 (3.5-30)	12.49 ± 5.50 (3.9-26)	0.879
**Short-axis diameter (mm)**	9.92 ± 4.47 (1.8-29)	9.90 ± 4.53 (1.8-29)	10.01 ± 4.23 (2.3-22)	0.856
**Mean CT attenuation (HU)**	-531.60 ± 138.07 (-801.5, -188)	-533.19 ± 138.15 (-790.9, -188)	-525.23 ± 138.59 (-801.5, -202.3)	0.669
**SD of CT attenuation (HU)**	96.72 ± 76.21 (4.5-1059)	97.88 ± 79.59 (4.5-1059)	92.07 ± 61.10 (16.1-317.1)	0.572
**Nodule shape**				0.980
Round or oval	165 (47.7)	132 (47.7)	33 (47.8)	
Irregular or polygonal	181 (52.3)	145 (52.3)	36 (52.2)	
**Tumor-lung interface**				0.448
Ill-defined	38 (11.0)	31 (11.2)	7 (10.1)	
Well-defined and smooth	207 (59.8)	168 (60.6)	39 (56.5)	
Well-defined but coarse	101 (29.2)	78 (28.2)	23 (33.3)	
**Spiculation (-)**	115 (33.2)	91 (32.9)	24 (34.8)	0.761
**Spiculation (+)**	231 (66.8)	186 (67.1)	45 (65.2)	
**Lobulation (-)**	128 (37.0)	106 (38.3)	22 (31.9)	0.327
**Lobulation (+)**	218 (63.0)	171 (61.7)	47 (68.1)	
**Cavity (-)**	343 (1)	274 (98.9)	69 (100)	0.387
**Cavity (+)**	3 (0)	3 (1.1)	0 (0)	
**Vacuole sign (-)**	92 (26.6)	72 (26.0)	20 (29.0)	0.616
**Vacuole sign (+)**	254 (73.4)	205 (74.0)	49 (71.0)	
**Air bronchogram (-)**	224 (64.7)	184 (66.4)	40 (58.0)	0.189
**Air bronchogram (+)**	122 (35.3)	93 (33.6)	29 (42.0)	
**Vascular convergence (-)**	155 (44.8)	127 (45.8)	28 (40.6)	0.432
**Vascular convergence (+)**	191 (55.2)	150 (54.2)	41 (59.4)	
**Intranodular vascular anomaly**				0.737
None	86 (24.9)	70 (25.3)	16 (23.2)	
Vessels entering with natural contour	99 (28.6)	79 (28.5)	20 (29.0)	
Vessels ingress into the nodule with dilated or distorted branches	161 (46.5)	128 (46.2)	33 (47.8)	
**Pleural retraction sign (-)**	243 (70.2)	193 (69.7)	50 (72.5)	0.651
**Pleural retraction sign (+)**	103 (29.8)	84 (30.3)	19 (27.5)	

The data are displayed as mean ± standard deviation (range) or number (%).

P value is derived from the t-test (two-tailed distribution, equal variance assumption) between training set and test set.

### The Result of Feature Selection

**Figure 4:**
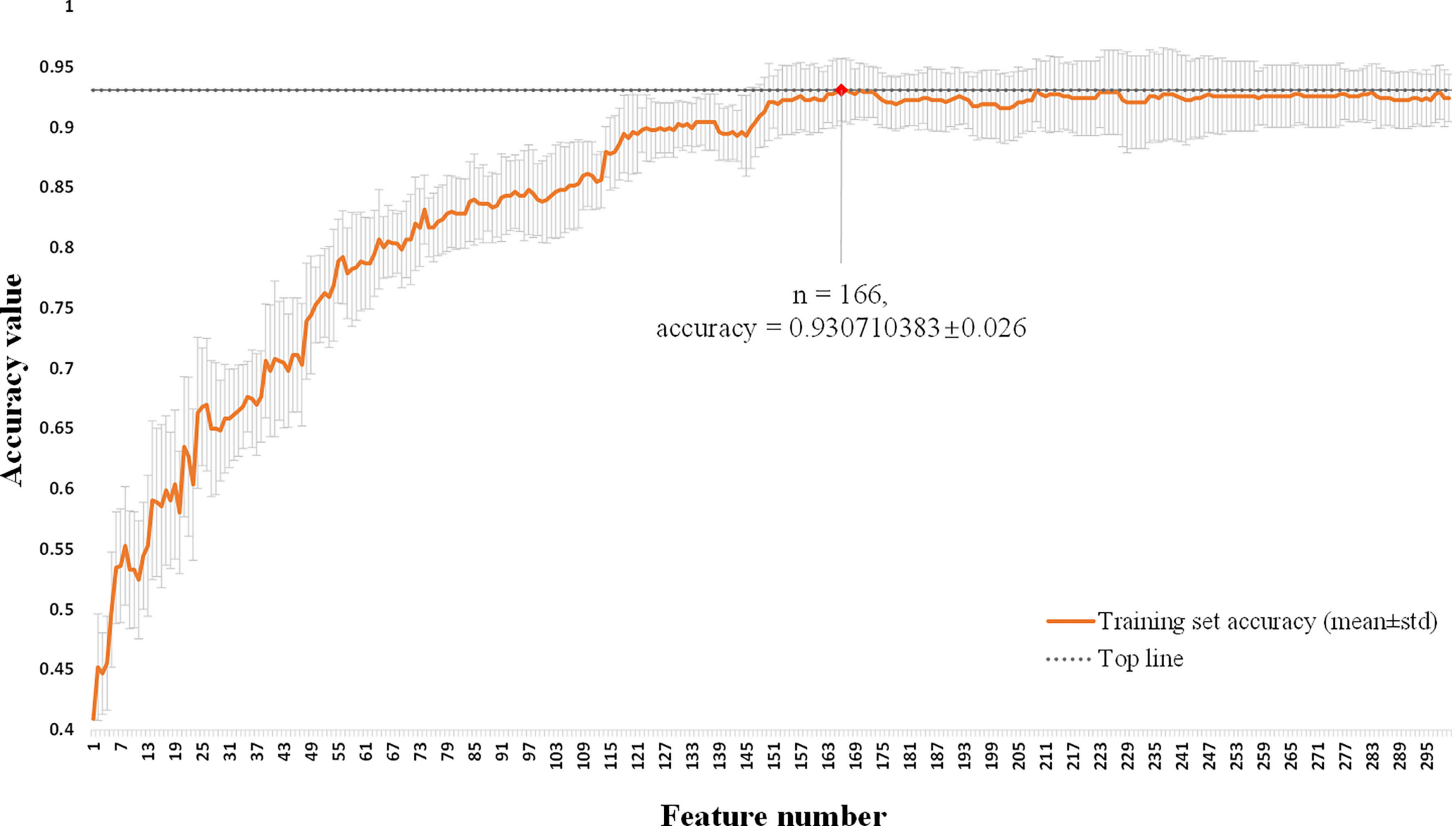
Feature selection of hybrid clinical-radiomics model using L2,1-norm minimization and logistic regression algorithm. The horizontal axis is the number of selected features (1 ≤ n ≤ 300). The vertical axis shows the corresponding average accuracy value of 10-fold cross-validation on the training set, and the gray area is the standard deviation. When the feature number is 166, the maximum accuracy value is obtained with the small standard deviation.

This multi-classification research used the L2,1-norm minimization and logistic regression algorithm to perform feature selection from the 1436 hybrid clinical-radiomics features on the training set. As shown in **Figure 4**, the average accuracy and standard deviation values corresponding to the number (1 ≤ n ≤ 300) of selected features were calculated by 10-fold cross-validation. It could be seen that when the number of selected features was 166, the highest accuracy value (0.931 ± 0.026) with a small standard deviation was obtained on the training set, so these 166 features could form an effective feature set for distinguish the degree of invasion for pGGNs. The detailed results of feature selection are shown in **Supplementary Table S1**.

### Analysis of Selected Features

**Figure 5:**
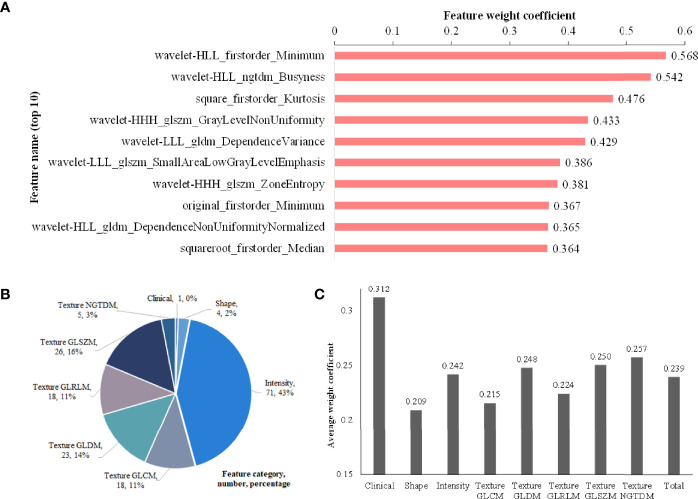
Feature analysis. **(A)** Histogram showing the weight coefficients of top 10 features within selected 166 hybrid features; **(B)** Description about category names, numbers and percentages of the 166 features; **(C)** The average weight coefficient of every category for the selected 166 features (There is only one clinical feature, so its *p* value cannot be calculated. No significant differences are found among other categories).

The weight coefficients of top 10 features are shown in **Figure 5A**, and the complete weight coefficients of all 166 features are listed in **Supplementary Table S2**. The three most important features with the highest weight coefficients are wavelet-HLL_firstorder_Minimum (0.568), wavelet-HLL_ngtdm_Busyness (0.542) and square_firstorder_Kurtosis (0.476).

**Figure 6:**
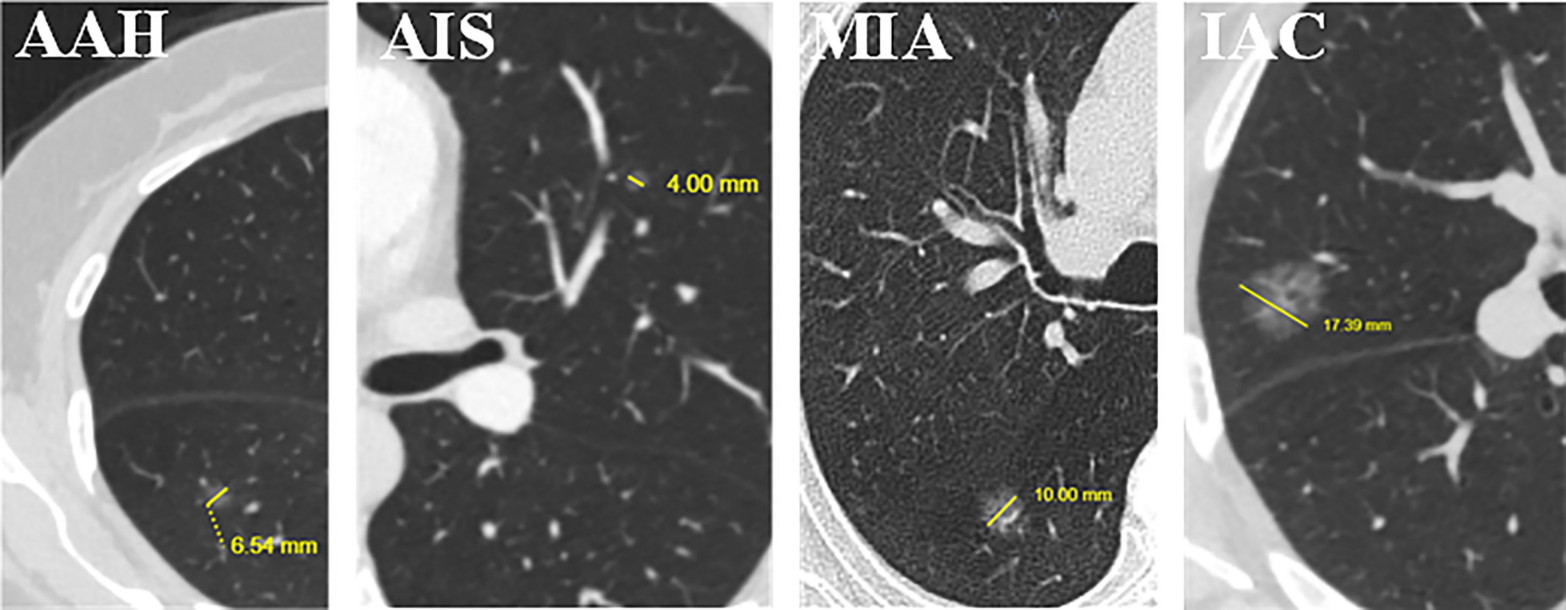
Examples of short-axis diameter (the vertical diameter of the longest diameter of the largest cross-section) (mm) for the four levels of invasion. We found that it is the only clinical feature in the 166 selected features used by the proposed hybrid-ensemble model. From left to right: atypical adenomatous hyperplasia (AAH), 6.54 mm; adenocarcinoma *in situ* (AIS), 4.00 mm; minimally invasive adenocarcinoma (MIA), 10.00 mm; invasive adenocarcinoma (IAC), 17.39 mm.

As shown in **Figure 5B**, the 166 selected features include 1 clinical feature (clinical short-axis diameter, ranked 23th) and 165 radiomics features. There are 4 (2%), 71 (43%) and 90 (55%) radiomics features from the tumor shape, intensity and texture features, respectively. Among the 90 tumor texture features, GLCM (n = 18), GLDM (n = 23), GLRLM (n = 18), GLSZM (n = 26) and NGTDM (n = 5) are all clearly present. We further analyze the importance of different categories of the selected 166 features through the average weight coefficient, as shown in **Figure 5C**. There is only one clinical feature, so its *p* value cannot be calculated. Among other radiomics categories, the features of intensity, texture GLDM, texture GLSZM and texture NGTDM show higher average weight coefficients than other feature categories, but no significant differences are found. Therefore, it can be considered that each feature category plays an important role for the multi-classification of invasiveness of pGGNs. The **Figure 6** shows the specific CT images of short-axis diameter with different invasion levels (AAH, AIS, MIA and IAC).

### Predictive Performance of Multi-Classification Models

**Figure 7:**
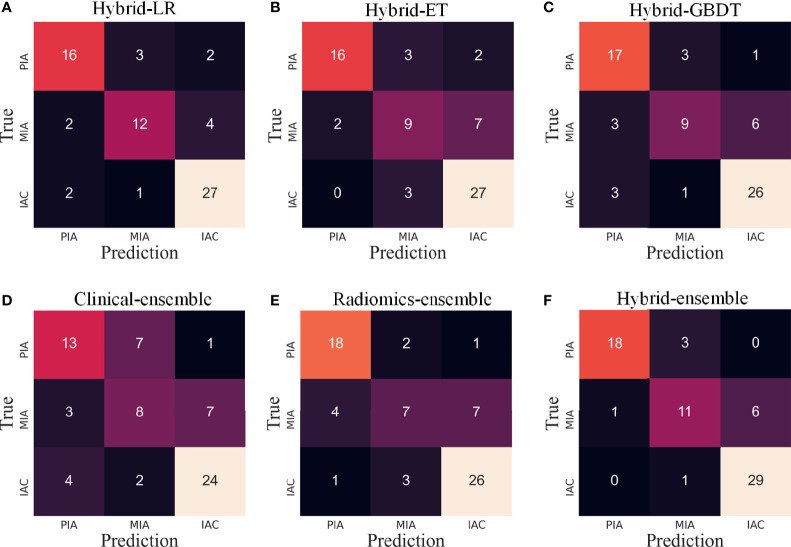
The confusion matrices of various models on the test set. LR, logistic regression; ET, extra trees; GBDT, gradient boosting decision tree. **(A–C)** Three algorithms (LR, ET, GBDT) with hybrid clinical-radiomics features; **(D–F)** Clinical model, radiomics model and hybrid clinical-radiomics model based on model ensemble of the three algorithms.

In this study, in order to distinguish among PIAs, MIAs and IACs, we respectively used three machine learning algorithms (LR, ET and GBRT) based on hybrid clinical-radiomics features to construct three multi-classification models. The three models were named hybrid-LR, hybrid-ET and hybrid-GBDT. We further integrated the results of three algorithms to obtain a hybrid-ensemble model through the model ensemble strategy. In addition, we also carried out the feature selection process from independent clinical features or radiomics features, as shown in **Figure S2**. Then we respectively constructed the clinical-ensemble model and radiomics-ensemble model based on the selected 20 clinical features and 275 radiomics features. Therefore, a total of 6 models were constructed, and their prediction confusion matrices on the test set are shown in **Figure 7**. It can be observed that the prediction performance of the six models for PIAs and IACs is better than MIAs, and the misclassified MIAs are more likely to be predicted as IACs than PIAs. The hybrid-ensemble model correctly classified more pGGNs on the test set compared to other five models. It could distinguish between the PIAs and IACs perfectly (There is no misclassification between the PIAs and IACs), and their wrong predictions were all classified as MIAs. For the hybrid-ensemble model, most of the misclassified cases (n = 6) of MIAs were predicted to be IACs, and only one MIA was incorrectly predicted as PIA.

For the 6 models, **Table 2** quantitatively lists their sensitivities of different invasion levels and overall classification accuracies on the training set and test set. Consistent with what is observed in **Figure 5**, the hybrid-ensemble model shows the strongest predictive performance among all 6 models. On the training set and test set, it obtained the F1-scores of 0.917 ± 0.024 and 0.824, and its accuracy values respectively were 0.918 ± 0.022 and 0.841. That indicated that the model ensemble strategy and hybrid clinical-radiomics features are important to improve the three-classification performance.

**Table 2:** The comparison of classification performance using different feature groups and algorithms.

Feature groups	Algorithms	Training set				Test set			
		Precision	Recall	F1-score	Accuracy	Precision	Recall	F1-score	Accuracy
Hybrid	LR	0.937 ± 0.033	0.933 ± 0.054	0.935 ± 0.041	0.931 ± 0.026	0.789	0.776	0.782	0.797
	ET	0.808 ± 0.103	0.805 ± 0.097	0.806 ± 0.100	0.807 ± 0.057	0.746	0.721	0.733	0.754
	GBDT	0.921 ± 0.057	0.908 ± 0.060	0.914 ± 0.058	0.909 ± 0.021	0.740	0.726	0.733	0.754
Clinical	Ensemble	0.795 ± 0.067	0.785 ± 0.049	0.790 ± 0.057	0.785 ± 0.029	0.624	0.621	0.622	0.652
Radiomics		0.865 ± 0.082	0.815 ± 0.037	0.839 ± 0.051	0.813 ± 0.067	0.710	0.704	0.707	0.739
Hybrid-ensemble model		0.917 ± 0.024	0.917 ± 0.024	0.917 ± 0.024	0.918 ± 0.022	0.836	0.812	0.824	0.841

LR, logistic regression; ET, extra trees; GBDT, gradient boosting decision tree. ± represents the standard deviation of the values in the 10-fold cross-validation.

## Discussion

In this study, we collected 27 clinical features and extracted 1409 radiomics features from each tumor three-dimensional VOI. After feature selection, we selected an effective feature set consisting of 166 features from the 1436 hybrid clinical-radiomics features. Based on the 166 hybrid features, we used three machine learning algorithms (LR, ET and GBDT) to construct three multi-classification models to distinguish the different invasion levels (PIA, MIA and IAC) of pGGNs. We further integrated the results of three algorithms to obtain a hybrid-ensemble model through the model ensemble strategy. Finally, we successfully constructed a multi-classification model to effectively distinguish different degrees of invasion for pGGNs. The proposed hybrid-ensemble model achieved the F1-score of 0.824 and an accuracy value of 0.841 on the independent test set, showing promising classification performance.

A precise diagnosis of the tumor invasion status is very important to guide individualized therapy in clinical practice. Early-stage lung adenocarcinoma often presents as GGN and has atypical features, which makes the differential diagnosis of the adenocarcinoma subtypes more difficult. Therefore, auxiliary identification by radiomics is necessary for early detection and prognosis of patients. Current researches mainly predicted the invasiveness of lung adenocarcinoma as invasive or non-invasive (2, 16–20, 30–33), and the multi-classification studies were rarely conducted to distinguish the degree of invasion in more detail. Our study attempted the three-classification of aggressive pGGNs, which is more meaningful.

Through the quantitative analysis of CT images, radiomics could objectively reflect both the attenuation and dispersion of gray level intensity, which might not be evident in direct visual assessments. Recent studies have shown that intensity and texture radiomics features are useful for predicting the invasiveness of lung adenocarcinoma presenting as GGNs (17, 18). This finding is consistent with our study, as the machine learning feature selection procedure selected 71 (43%) intensity and 90 (55%) texture features to establish the hybrid-ensemble model. In addition, in total 166 features were selected, of which only one clinical feature (short-axis diameter, ranked 23th). It meant that the short-axis diameter was the most important parameter for the invasive classification of pGGNs among the 27 clinical features. We found that in general, lung nodules with large short-axis diameter have the higher degree of invasion. Compared with the maximum long-axis diameter, the short-axis diameter implies a longer diameter in the vertical direction, which represents more nodule size information. Previous studies (16, 30–32) found that the size (usually quantified by area) of the nodule is an important parameter for assessment of lung adenocarcinoma invasiveness, which is somewhat consistent with short-axis diameter. However, we believe that the short-axis diameter may be more advantageous in some respects, as it contains information about the shape of the nodule in addition to its size.

Previous studies tried hybrid clinical-radiomics features to build radiomics models, and the results showed that this is effective for more accurate classification (2, 20, 33). Our study also demonstrated this, using the joint 1436 features make the hybrid-ensemble model perform better than clinical-ensemble and radiomics-ensemble. In addition, we further introduced the model ensemble strategy, which has not been tried by researchers before, and our model comparison experiments showed that this strategy is also very effective. For the proposed hybrid-ensemble model, the classification performance of MIAs is slightly low, similar to the fact that it is more difficult for clinicians to distinguish MIAs in actual clinical diagnosis, which may be because MIAs are of the intermediate degree of invasion. We further found that most of the misclassified cases of MIAs were predicted to be IACs, which means that these two grades were more difficult to be distinguished. In addition, the hybrid-ensemble model had no misclassification to distinguish between IACs and PIAs, showing its potential clinical application value.

This study has several limitations. First of all, this is a single-center retrospective study, and a multi-center study is better to be conducted to further evaluate the model performance. Second, relying only on the radiologists to manually delineate and segment the region of interest is more time-consuming and subjective, and reliable and automatic methods are essential to simplify the complex procedures.

In conclusion, this study used the short-axis diameter parameter and 165 radiomics features to construct a multi-classification model for precisely predicting the invasiveness of lung adenocarcinoma with pGGNs. We found that short-axis diameter was the most important parameter among 27 clinical features. The hybrid-ensemble model based on hybrid clinical-radiomics features and model ensemble strategy had better predictive performance, and could have a promising clinical application value.

## Data Availability Statement

The raw data supporting the conclusions of this article will be made available by the authors, without undue reservation.

## Ethics Statement

Written informed consent was obtained from the individual(s) for the publication of any potentially identifiable images or data included in this article.

## Author Contributions

FS and LS made major contributions to the research and writing of manuscripts.TX, YF, and XS assisted in the research and data analysis. PZ and TZ provided some code suggestions. ZZ assisted in data curation and processing. WS and GZ provided the supervisions of the entire study and made contributions to review and editing of manuscripts. All authors contributed to the article and approved the submitted version.

## Funding

This work was partially supported by the Beijing Natural Science Foundation (7202102), the National Natural Science Foundation of China (61871022), the Fundamental Research Funds for Central Universities, the 111 Project (B13003), and the 2021 SKY Imaging Research Fund of Chinese International Medical Exchange Foundation (Z-2014-07-2101).

## Conflict of Interest

The authors declare that the research was conducted in the absence of any commercial or financial relationships that could be construed as a potential conflict of interest.

## Publisher’s Note

All claims expressed in this article are solely those of the authors and do not necessarily represent those of their affiliated organizations, or those of the publisher, the editors and the reviewers. Any product that may be evaluated in this article, or claim that may be made by its manufacturer, is not guaranteed or endorsed by the publisher.
