# Supplementary material for: A Multi-Classification Model for Predicting the Invasiveness of Lung Adenocarcinoma Presenting as Pure Ground-Glass Nodules
Source: Front Oncol. 2022 Apr 28;12:800811. doi: 10.3389/fonc.2022.800811 (PMC9096139; doi:10.3389/fonc.2022.800811)
Supplement: Supplementary file 1 [file DataSheet_1.docx]

Supplementary Material


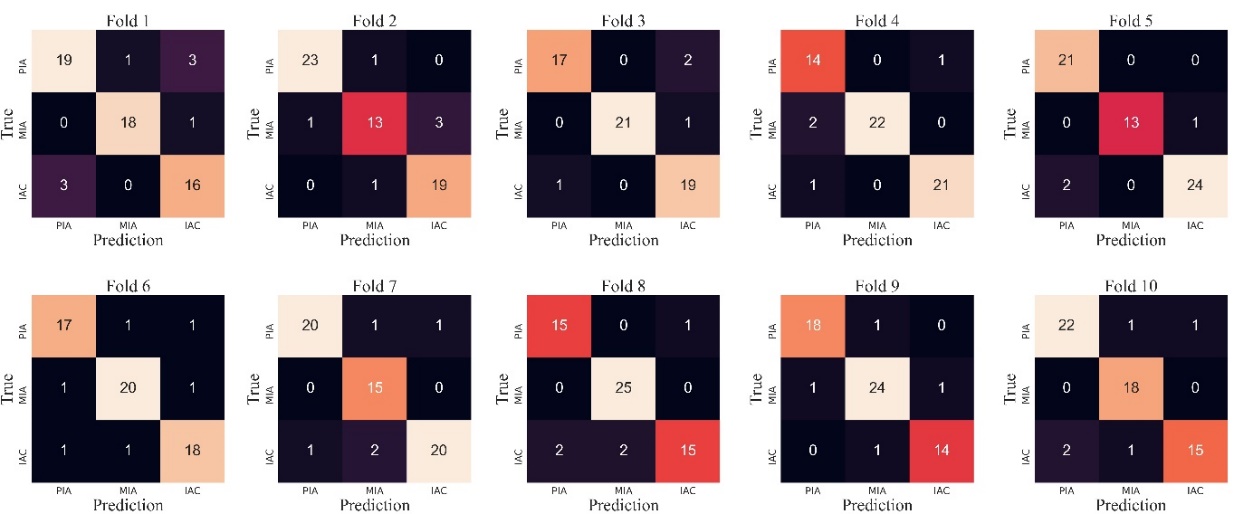


**Fig. S1** Confusion matrices on the training set for the ensemble model based on hybrid clinical-radiomics features


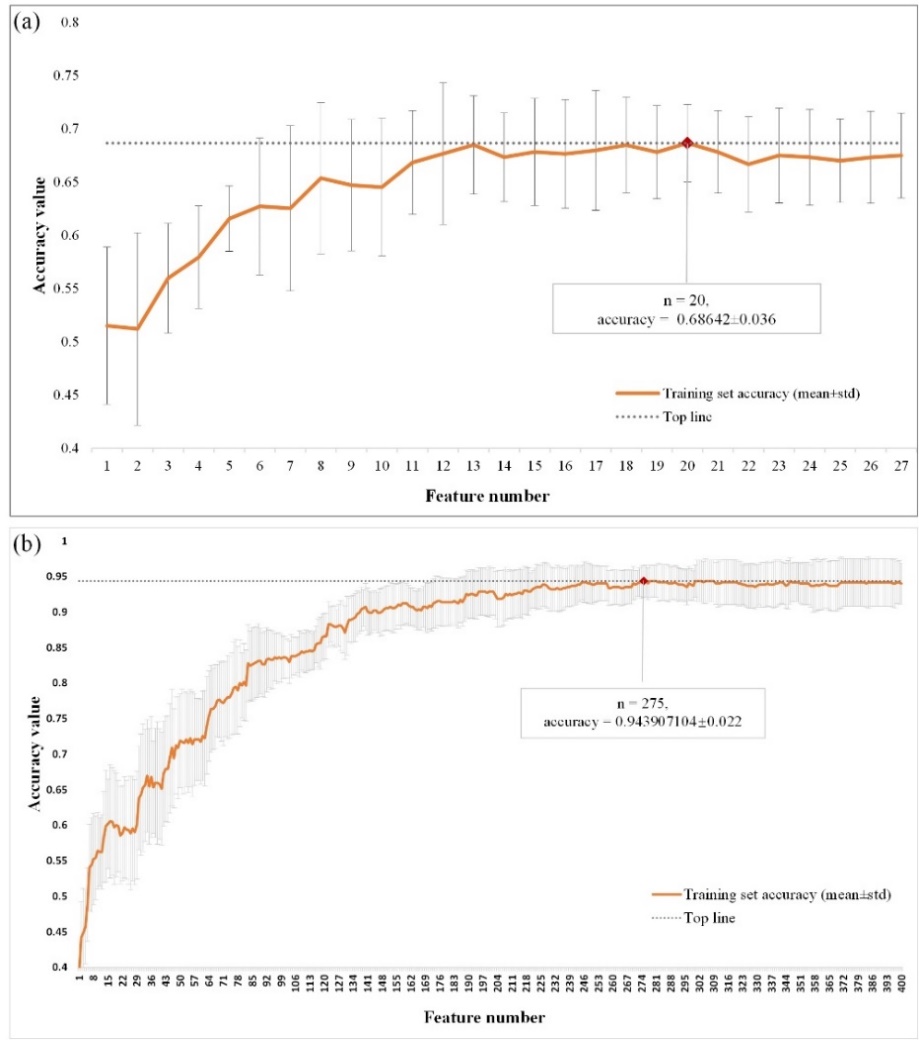


**Fig. S2** Feature selection using L2,1-norm minimization and logistic regression algorithm. The horizontal axis is the number of selected features. The vertical axis shows the corresponding average accuracy value of 10-fold cross-validation on the training set, and the gray area is the standard deviation. (a) Feature selection of the clinical model. When the feature number is 20, the maximum accuracy value is obtained with the small standard deviation. (b) Feature selection of the radiomics model. When the feature number is 275, the maximum accuracy value is obtained with the small standard deviation

**Table S1** Specific description of feature selection for hybrid clinical-radiomics features, clinical features and radiomics features

(Supplementary Table S1. Feature selection results .xlsx)

**Table S2** Specific description of the 166 selected hybrid clinical-radiomics features and corresponding feature weight coefficients after feature selection

(Supplementary Table S2. The feature set consisting of 166 selected features .xlsx)

**Table S3** Hyperparameters of four algorithms after fine-tuning based on the hybrid clinical-radiomics features

| Algorithms | Model hyperparameters |
| --- | --- |
| Logistic regression | random_state= 42, penalty='l2', solver='lbfgs', C=0.7, class_weight=None, max_iter=100, multi_class='auto' |
| Extra trees | n_estimators=35, min_samples_split=4, random_state=42, max_depth=6 |
| GBDT | n_estimators=57, learning_rate=0.1, max_depth=8, num_leaves=19, boosting_type='gbdt', objective='multiclass', num_class=3, metric='multi_error', random_state=80 |
| Ensemble | estimators=[('GBDT', GBDT), ('ExtraTrees', ExtraTrees), ('LogisticRegression', LogisticRegression)], voting='hard', weights=[4,3,5] |

**Table S4.** The results of 10-fold cross-validation on the training set

| Fold | Type | Accuracy | Sensitivity | Samples | Fold | Accuracy | Sensitivity | Samples |
| --- | --- | --- | --- | --- | --- | --- | --- | --- |
| 1 | PIA | 0.869 | 0.826 | 23 | 6 | 0.902 | 0.895 | 19 |
|  | MIA |  | 0.947 | 19 |  |  | 0.909 | 22 |
|  | IAC |  | 0.842 | 19 |  |  | 0.900 | 20 |
| 2 | PIA | 0.902 | 0.958 | 24 | 7 | 0.917 | 0.909 | 22 |
|  | MIA |  | 0.765 | 17 |  |  | 1.000 | 15 |
|  | IAC |  | 0.950 | 20 |  |  | 0.870 | 23 |
| 3 | PIA | 0.934 | 0.895 | 19 | 8 | 0.917 | 0.938 | 16 |
|  | MIA |  | 0.955 | 22 |  |  | 1.000 | 25 |
|  | IAC |  | 0.950 | 20 |  |  | 0.789 | 19 |
| 4 | PIA | 0.934 | 0.933 | 15 | 9 | 0.933 | 0.947 | 19 |
|  | MIA |  | 0.917 | 24 |  |  | 0.923 | 26 |
|  | IAC |  | 0.955 | 22 |  |  | 0.933 | 15 |
| 5 | PIA | 0.951 | 1.000 | 21 | 10 | 0.917 | 0.917 | 24 |
|  | MIA |  | 0.929 | 14 |  |  | 1.000 | 18 |
|  | IAC |  | 0.923 | 26 |  |  | 0.833 | 18 |
